# Are Physics Forceps Less Traumatic than Conventional Forceps for Tooth Extraction? A Systematic Review and Meta-Analysis of Randomized Controlled Trials

**DOI:** 10.3390/dj10020021

**Published:** 2022-01-31

**Authors:** Ashutosh Kumar Singh, Nikita Khanal, Nisha Acharya, Dinesh Rokaya, Md Riasat Hasan, Takashi Saito

**Affiliations:** 1 Department of OMFS, TU Teaching Hospital, MMC, IOM, Kathmandu 44600, Nepal; dr.ashutosh@iom.edu.np; 2 Dental Surgeon, Ek EK Paila Foundation, Kathmandu 44600, Nepal; drnikitakhanal@gmail.com; 3 Department of Conservative Dentistry and Endodontics, TU Teaching Hospital, MMC, IOM, Kathmandu 44600, Nepal; menishaacharya@gmail.com; 4 Department of Clinical Dentistry, Walailak University International College of Dentistry, Walailak University, Bangkok 10400, Thailand; dineshrokaya115@hotmail.com; 5 Division of Clinical Cariology and Endodontology, Department of Oral Rehabilitation, School of Dentistry, Health Sciences University of Hokkaido, Ishikari 061-0293, Japan; t-saito@hoku-iryo-u.ac.jp

**Keywords:** tooth extraction, dental instruments, complications, systematic review, meta-analysis

## Abstract

This systematic review and meta-analysis studied the clinical outcomes with physics forceps compared to those with conventional forceps for closed dental extraction. A systematic literature search was performed to identify all the published randomized clinical trials that compared the relevant clinical outcomes with physics forceps to those with conventional forceps for closed dental extraction. A total of 11 studies were included. The adverse events were significantly lower with physics forceps (*n* = 48) compared to with conventional forceps (*n* = 120), with an odds ratio of 0.42 [0.25, 0.70], Z = 3.78 (*p* = 0.0002), and I^2^ = 21%. There were statistically significant differences in the incidence of GL (*p* = 0.04), and tooth or root fracture (*p* = 0.0009). Operating time was significantly lower in physics forceps than that of conventional forceps, mean difference (−20.13 (−30.11, −10.15)), Z = 3.78 (*p* = 0.0001), I^2^ = 79%. The available evidence is limited by a high risk of bias and low evidence certainty. Based on the current evidence, physics forceps might be better than the conventional extraction forceps in terms of the extraction duration, pain after extraction, trauma to both hard and soft tissue, and complications. Physics forceps are newer instruments that have not yet been introduced in the teaching of dental graduates. The introduction of physics forceps can be time saving, less invasive and reduce post-extraction complications.

## 1. Introduction

Dental extraction is one of the most common procedures performed by dentists. Indications for extraction are impaction, tooth or root fracture, caries, or periodontal disease [1]. Using ideal principles of extraction permits the effective, efficient, and safe removal of teeth, reducing complications. The lack of proper instrumentation and physics principles can result in a long duration of extraction and iatrogenic trauma to the patient, and fatigue and injury to the clinician [1,2,3]. Atraumatic extraction is always preferred, especially in the case of a planned immediate implant placement, predictable orthodontic tooth movement, or in patients with compromised bone quality and quantity [4]. Immediate implant placement requires fully intact osseous and soft tissue, which can be achieved by less-traumatic tooth extraction [5,6,7]. Traditional forceps and elevators often result in soft- and hard-tissue damage to the loss of the buccal bony plate and interdental bone crest [8,9].

A variety of new instruments and techniques have been introduced for atraumatic tooth extractions. A powered periotome and newer implant drills are useful for immediate or delayed implant placement [6,10,11,12]. The physics forceps (GoldenDent, Roseville, MI, USA) provides a mechanical advantage to extract teeth reducing the use of excessive force [8]. This advantage minimizes root or alveolar bone fractures, and helps to preserves the surrounding bone. It has a bumper in the buccal vestibule and a thin beak on the lingual aspect of the tooth, and thus utilizes a first-class lever action by applying constant pressure that slowly elevates the root from the socket [13]. This causes the release of hyaluronidase in the periodontal-ligament (PDL) space, which results in the gradual release of the PDL, and the tooth becomes mobile and can be easily removed [14]. Multiple studies published comparative results of physics forceps compared to those of the conventional forceps [15,16,17,18,19,20,21,22,23,24,25]. A recent narrative systematic review also reported adverse events and operative time, comparing the physics and conventional forceps but a meta-analysis was not performed [26].

The objectives of this meta-analysis was to update the evidence comparing these two instruments and perform quantitative analysis of clinically important outcomes to present an absolute measure of effect. Additionally, a summary of findings table was constructed on the basis of the GRADE approach to ascertain the certainty of evidence, and provide a clinical recommendation based on the evidence.

## 2. Materials and Methods

### 2.1. Literature Search

This systematic review was registered on PROSPERO (CRD42021268530). Electronic literature search was performed on databases PubMed/Medline, Web of Science, U.S. clinical-trial registries (ClinicalTrials.gov), Google Scholar, and proceedings from major scientific meetings published by May 2020 in English language. The ClinicalTrials.gov website was searched for unpublished trials. The detailed search strategy is presented in Appendix A.

### 2.2. Study Selection

PRISMA reporting guidelines were followed. In addition, the PICOS principle that was used to select studies is provided in Table 1. Uncontrolled trials, reviews, letters to editors, case series and case reports, retrospective and prospective cohort studies, updates, interviews, commentaries, and animal studies were excluded.

### 2.3. Quality Assessment

The qualities of the included studies were assessed using criteria from the Cochrane Collaboration’s Handbook (*Cochrane Handbook for Systematic Reviews of Interventions Version 5.1.0*, Higgins and Greene) using the ROB 2 tool [27,28].

### 2.4. Statistical Analysis

This meta-analysis was performed with the Cochrane RevMan software version 5.4. Dichotomous nominal data with odds ratios and continuous scale data as mean differences at a 95% confidence interval were evaluated. In this meta-analysis, Mantel–Haenszel fixed effect models were established for dichotomous data and inverse variance random-effect models for continuous data. The I^2^ statistical test was conducted to study statistical heterogeneity. The evidence was graded, and certainty was derived with the GRADEpro Guideline Development Tool. When significant heterogeneity was encountered, visual inspection of the forest plot and formal sensitivity analysis were performed to explain the heterogeneity. Publication bias and the small-study effect were analysed with a funnel plot and formal statistical tests if required.

## 3. Results

**Figure 1:**
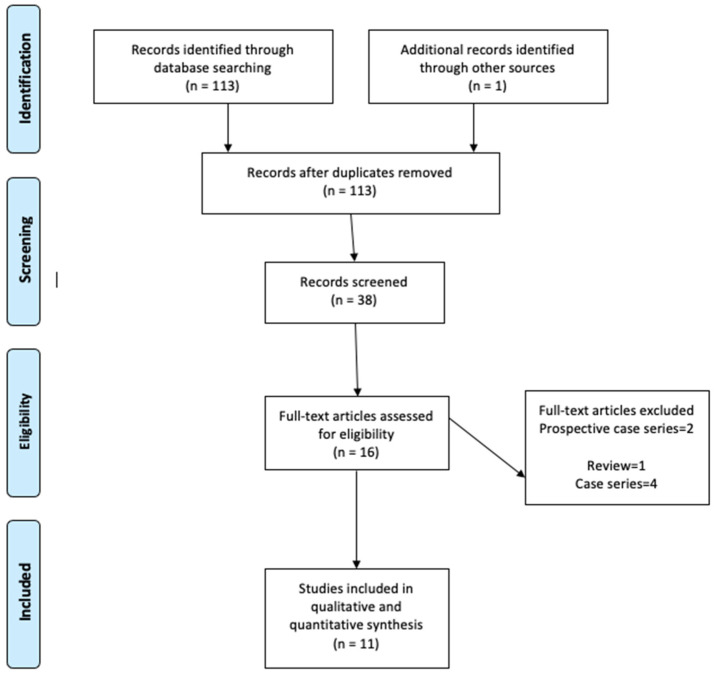
Preferred Reporting Items for Systematic Reviews and Meta-Analyses (PRISMA) flow diagram of trial selection.

We identified 11 articles that met our inclusion criteria (number of extracted teeth = 1028, physics forceps = 514, conventional forceps = 514) [15,16,17,18,19,20,21,22,23,24,25]. A diagram for the study selection is presented in Figure 1. The study characteristics, and results of primary and secondary outcomes are presented in Table 2.

**Figure 2:**
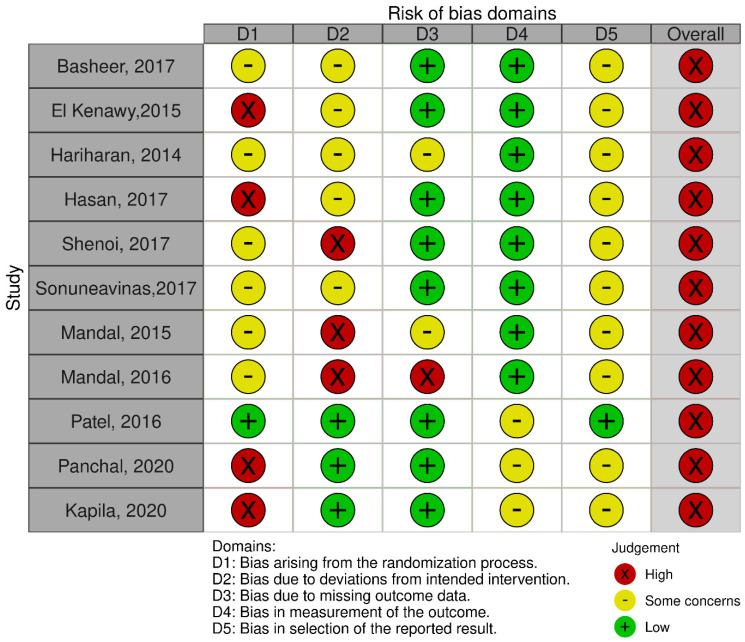
Risk of bias within individual trials.

**Figure 3:**
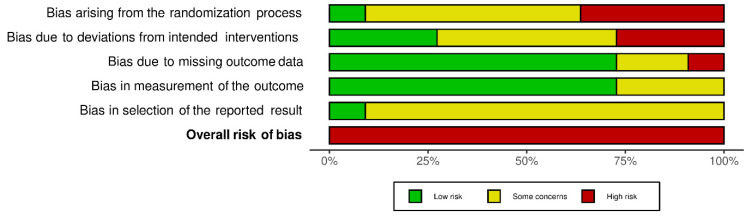
Risk of bias across included trials.

All trials were performed in India except for one, which was conducted in Iraq. The included studies were published between 2014 and May 2020, with the sample size ranging from 14 to 200. Five trials [16,17,19,20,25] used the split-mouth technique to compare between two groups, while six other studies [15,18,21,22,24] used the parallel group-randomization technique. The randomization method was computer-generated in four studies [18,21,22,23], coin tossing in four studies [17,19,20], and there were no details of randomization in three studies [15,16,24]. Three studies included extractions of canines and incisors, eight of premolars, and four of molars. Three studies included more men, two studies had more women, one study had equal gender distribution, and five studies did not mention gender distribution. Buccal cortical plate fracture (BCPF), amount of soft-tissue or gingival loss (GL), post-operative pain, time taken for extraction, and crown or tooth and root fractures were the most common outcomes measured in most of the studies, followed by soft-tissue healing after extraction. Other outcomes that were measured were bleeding, the ease of the technique, the volume of analgesics taken, bone loss, dry socket, and infections. All the trials had a high risk of bias. The risk of bias in individual studies is shown in Figure 2. The risk of bias across the included studies is shown in Figure 3.

**Figure 4:**
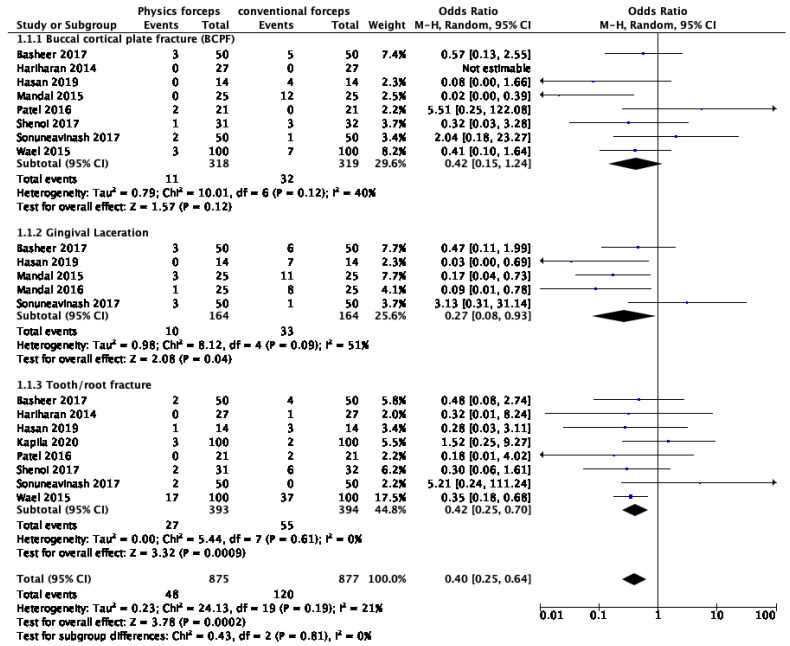
Meta-analysis of adverse events organised into subgroups (buccal cortical plate fracture, gingival laceration, and tooth or root fracture).

The results of the meta-analysis of adverse events are presented in Figure 4, organised into subgroups (BCPF, gingival laceration, and tooth or root fracture). Adverse events were statistically significantly lower with the physics forceps (*n* = 48) compared to with the conventional forceps (*n* = 120), with odds ratio 0.42 (0.25, 0.70), Z = 3.78 (*p* = 0.0002), and I^2^ = 21%. There was a significant difference in the incidence of GL (*p* = 0.04), and tooth or root fracture (*p* = 0.0009).

**Figure 5:**
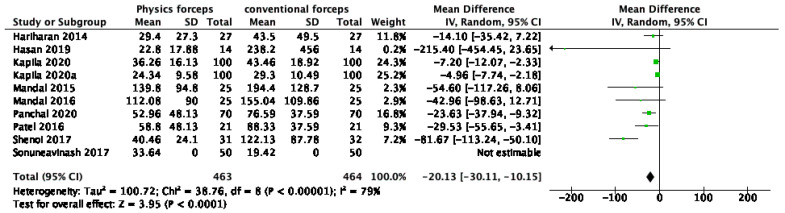
Forest plot for the meta-analysis of operative time.

The meta-analytical results of operative time are presented in Figure 5. Operating time was statistically significantly lower in physics forceps than that in conventional forceps; mean difference (−20.13 (−30.11, −10.15)), Z = 3.78 (*p* = 0.0001), and I^2^ = 79%.

**Figure 6:**
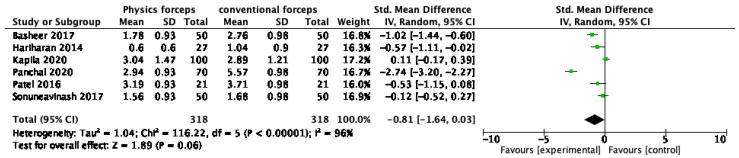
Forest plot for the meta-analysis of pain on first post-operative day.

**Figure 7:**
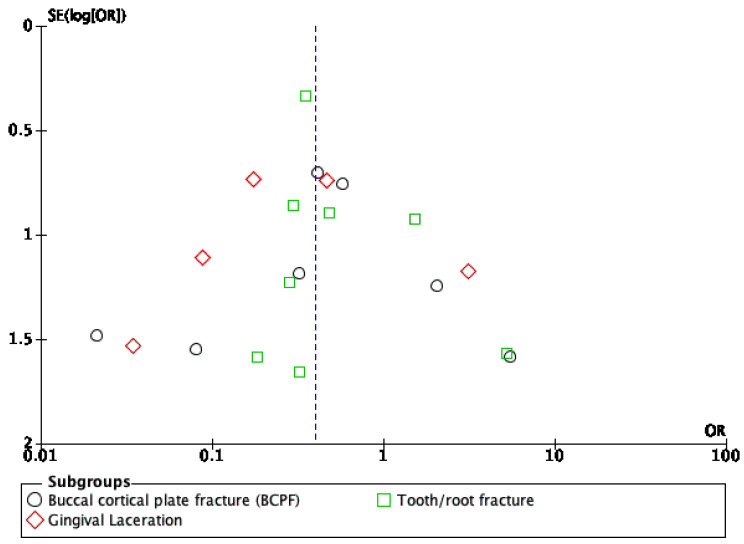
Funnel-plot analysis for publication bias.

The results of the meta-analysis of pain on the first post-operative day are presented in Figure 6. The overall pain after extraction was better with physics forceps than that with conventional forceps (standardized mean difference = −0.81 (−1.64, 0.03)), but there was no significant difference (Z = 3.78 (*p* = 0.06) and I^2^ = 96%). The funnel plot presented in Figure 7 does not show significant asymmetry; hence, the probability of publication bias is lower. Formal statistical tests were not conducted because of the small number of studies consistently reporting on the same outcome.

## 4. Discussion

There was a significant reduction in adverse events (77 fewer per 1000) with the physics forceps compared to with the conventional forceps, which can be understood as 8 fewer adverse events per 100 extractions. Individually, the reduction in GL and tooth/root fracture are statistically significant with the physics forceps, but the difference did not achieve statistical significance for BCPF. This is a significant reduction in adverse clinical events with which to recommend a change in practice from regularly using the conventional to the physics forceps. The reduction in BCPF (56 fewer/1000), GL (138 fewer/1000), and tooth or root fracture (76 fewer/1000) is very promising and supports using the physics forceps over the conventional forceps. The evidence has low certainty for all the outcomes. Even the mean difference in operative time and pain on VAS favours the physics forceps with low-to-moderate certainty. The evidence for the outcomes of BCPF, GL and tooth or root fracture was very low because of inconsistencies in the measurement and the probability of selective reporting, and studies have a high risk of bias, used in the GRADE approach for the evaluation of evidence certainty (Table 3).

The physics forceps design reduces the incidence of root fractures and maintains the buccal cortical plate. This is also necessary for the success of an immediate dental implant. The forceps is an innovative extraction instrument that can perform difficult extractions without the need to reflect a flap with predictable results [15,18,23]. The use of physics forceps helps in the prevention of marginal-bone and soft-tissue loss, maintaining gingival and periodontal integrity with fewer complications. In this study, for adverse events, the samples had low heterogeneity (I^2^ = 21%). For the operating time, the samples showed moderate-to-high heterogeneity (I^2^ = 79%). Sensitivity analysis by removing the results from Hasan and Shenoi brought the heterogeneity down to 58%. Similarly, for the pain after extraction, the samples showed high heterogeneity (I^2^ = 96%). Sensitivity analysis by selectively removing the studies did not resolve the heterogeneity. This high heterogeneity may be because of the subjective evaluation of pain on VAS. Although VAS is traditionally treated as a continuous outcome, it is essentially not a numerical scale, and is based on subjective perception. Similarly, various other comparative studies of physics versus conventional forceps in orthodontic extractions also showed better results for physics forceps [17,19,20,25]. Physics forceps showed better results in the extraction of premolars and molars [18,21,24].

Extractions using physics forceps are less invasive than those with conventional forceps, and can be considered a reliable method, requiring significantly less comparative intraoperative time. The physics forceps requires a learning curve, but gives the clinician the unique opportunity to atraumatically undertake conventionally difficult extractions in order to maintain alveolar height, thus facilitating immediate prosthetic rehabilitation. Unlike the use of conventional forceps in training in dental schools, the physics forceps is a novel instrument, however, the learning of wrist movements and the direction of application of force is not difficult for the users of conventional forceps. The physics forceps is more efficient in reducing operating time, and preventing marginal-bone and soft-tissue loss in orthodontically indicated premolar extractions, with comparable clinical outcomes to those of the conventional forceps, associated with few complications.

## 5. Conclusions

Physics forceps are newer instruments that have not yet been introduced in the teaching of dental graduates. Physics forceps compare well to the conventional extraction forceps in terms of extraction duration, pain after extraction, and lesser trauma to both hard and soft tissue, with fewer complications. We recommend the introduction of physics forceps in regular clinical use and the training of dental graduates. Further trials are required, especially in other population groups, to evaluate the physics forceps as a better alternative and successor to the conventional dental-extraction forceps.

## Figures and Tables

**Table 1:** PICOS (patients, intervention, comparison, outcomes, and study design) criteria to select studies.

	Inclusion Criteria	Exclusion Criteria
(P) Patients or population	Extraction of any permanent tooth in healthy patients.	Extraction of deciduous teeth, teeth associated with pathology
(I) Intervention	Physics forceps	
(C) Comparator or control group	Conventional forceps	
(O) Outcomes	Primary outcome: BCPF, GL, tooth or root fracture. Secondary outcomes: operative time, pain after extraction	
(S) Study design	Studies in humans RCTs.	Uncontrolled clinical trials, prospective and retrospective comparative studies, reviews, case series, and reports.

BCPF = buccal cortical plate fracture, GL = gingival lacerations, RCT = randomized control trials.

**Table 2:** Study characteristics.

Author, Country, Year	Study Design	Sample Size, PF CF	Age, Sex	Teeth Extracted	Outcome	Results
Basheer, India, 2017 [24]	Parallel group RCT	Patients: 100 Teeth: 100 PF: 50 CF: 50	NG	Maxillary molars	BCPF GL Tooth/root fracture, socket healing, bleeding from socket, post-operative pain (1–5 days).	BCPF: NSD (*p* = 0.715). GL: NSD (*p* = 0.487). Tooth fracture: NSD (*p* = 0.678). Healing: SSD (*p* = 0.002). Bleeding: SSD (*p* = 0.001). Pain: SSD on Days 1 (*p* < 0.001) and 2 (*p* < 0.001), and NSD on Days 3 (*p* = 0.374), 4 (*p* = 0.543), and 5 (*p* = 1.00).
El-Kenawy and Ahmed, India, 2015 [15]	Parallel group RCT	Patients: 200 Teeth: 200 PF: 100 CF: 100	Age: Mean: PF: 42.6 ± 15.9 (SD) CF: 41.6 ± 15.1 (SD) Gender: Male: 136 Female: 64	Any tooth	BCPF Crown fracture, root fracture.	BCPF: NSD (*p* = 0.19). Crown fracture: SSD (*p* = 0.04). Root fracture: SSD (*p* = 0.027).
Hariharan et al., India, 2014 [20]	Split-mouth RCT	Patients: 27 Teeth: 54 PF: 27 CF: 27	Age: Mean: 16 Range: 11–23 Gender: Male: 12 Female:15	Maxillary premolars	BCPF Tooth/root fracture, dry socket delayed healing, acute infection, post-operative pain after 1, 3, and 7 days.	BCPF: NSD (*p* = NG) Tooth/Root fracture: NSD (*p* = NG) Delayed healing, dry socket, and acute infection: NSD (*p* = NG) Pain: SSD on Day 1 (*p* = 0.03), no pain on Days 3 and 7. Extraction Time: NSD (*p* = 0.204)
Hasan, Iraq, 2017 [23]	Parallel group RCT	Patients: 14 Teeth: 28 PF: 14 CF: 14	Age: Mean: 40.7 Range 16–65 Gender: Male: 8 Female: 6	Mandibular incisors, canines, and premolars	BCPF GL Tooth/root fracture, extraction time.	BCPF: NSD (*p* = 0.098) GL: SSD (*p* = 0.006). Crown fracture: NSD (*p* = 0.222). Root fracture: NSD (*p* = 1.00). Time: SSD (*p* = 0.01).
Shenoi et al., India, 2017 [18]	Parallel group RCT	Patients: 64 Teeth: 64 PF: 31 (1 excluded) CF: 32	Age Mean: 44.11 Range: 21–70 Gender: Male: 30 Female: 30	Maxillary molars	BCPF Root fracture, delayed healing, dry-socket infection pain after 1, 3, and 7 days, extraction time.	BCPF: NSD (*p* = 0.612). Root fracture: NSD (*p* = 0.129). Delayed healing, dry socket, and infection: NSD (*p* = not calculated, *p* = 1.000, and *p* = 0.150, respectively). Pain: SSD on post-operative Days 1 and 3. (*p* = 0.0007 and *p* = 0.0008, respectively) and no pain on Day 7. Time: SSD (*p* < 0.001).
Sonun Avinash et al., India, 2017 [25]	Split-mouth RCT	Patients: 50 Teeth: 100 PF: 50 CF: 50	Range: 14–25 NG	Maxillary premolars	BCPF GL Tooth/ root fracture, bleeding, soft-tissue healing after 7, 14, and 21 days, pain after 1–7 days, ease of technique, extraction time.	BCPF: NSD (*p* = 0.55) GL: NSD (*p* = 0.30) Tooth or root fracture: NSD (*p* = 0.15). Bleeding: SSD (*p* < 0.001). Post-operative healing after 7 days: NSD (*p* = 0.21). Pain: NSD on Days 1 to 4 (*p* = 0.07–0.97) and no pain on Days 5–7. Techical ease and learning curve: NSD (*p* = 0.26) Time: SSD (*p* = not calculated)
Mandal et al., India, 2015 [21]	Parallel group RCT	Patients: 50 Teeth: 50 PF: 25 CF: 25	>14 NG	Mandibular molars	BCPF, GL, Pain after 3 and 7 days, and extraction time.	BCPF: SSD (*p* = 0.001). GL: SSD (*p* = 0.032). Pain: SSD on day 3 (*p* = 0.035) Extraction Time: SSD (*p* = not calculated)
Mandal et al., India, 2016 [22]	Parallel group RCT	Patients: 50 Teeth: 50 PF: 25 CF: 25	>14 NG	Mandibular incisors, canines and premolars	BCPF, GL, tooth fracture extraction time.	BCPF: NG GL: SSD (*p* = 0.032). Tooth fracture: NG. Time: SSD (*p* = not calculated)
Patel et al., India, 2016 [19]	Split- mouth RCT	Patients: 11 Teeth: 42 PF:21 CF:21	Age: Mean: 19.4 Range: 14–23 years Gender: Male: 7 Female:4	Maxillary and mandibular premolar teeth	BCPF, GL, ease of technique, pain, extraction time, other complications.	BCPF: PF 4.76%, CF: none Root fracture: PF: 4.76%, CF: none GL: SSD (*p* = 0.035). Marginal bone loss: SSD (*p* = 0.037). Time: SSD (*p* = 0.019) Techncal ease and learning curve: NSD (*p* = NG) Pain: NSD (*p* = NG)
Kapila et al., India, 2020 [17]	Split mouth RCT	Patients: 50 Teeth: 200 PF: 100 CF: 100	Age Mean: 17.6 Range: 14–25 Gender Male: 14 Female:36	Maxillary and mandibular premolars	Time, BCPF, GL, volume of analgesics, healing post-operative pain on the day, and 1, 3, and 7 days after extraction, complications.	Time: SSD (*p* = 0.001) Pain: NSD Day of extraction (*p* = 0.927), 1 day after extraction (*p* = 0.513), 3 days after (*p* = 0.349), 7 days after (*p* = 0.445) Volume of analgesics: not significant (*p* = 0.522) BCPF: no significant difference (*p* = NG) GL: NSD (*p* = NG) Tooth, root, or alveolus fracture: No event in any group
Panchal et al., India, 2020 [16]	Split mouth RCT	Patients: 35 Teeth: 140 PF: 70 CF: 70	Range: 18–40 Gender: NG	Maxillary and Mandibular Premolars	Time, BL, GL, success score and pain.	Time: SSD (*p* = 0.001) GL: SSD (*p* = 0.001). Bone loss: SSD (*p* = 0.001). Success score: Mean score: PF: 5 (95.92%) CF: 3.9 (91.84%)

RCT = randomized control trials, PF = physics forceps, CF = conventional forceps, GF = gingival lacerations, BCPF = buccal cortical plate fracture, BL = bone loss, GL = gingival lacerations, NG = not given, NSD = no significant difference, SSD = statistically significant difference.

**Table 3:** GRADE summary of findings.

Physics Forceps Compared to Conventional Forceps for Closed Tooth Extraction					
Patient or population: closed tooth extraction Setting: Intervention: physics forceps Comparison: conventional forceps					
Outcomes	N° of participants (studies) Follow up	Certainty of the evidence (GRADE)	Relative effect (95% CI)	Anticipated absolute effects	
				Risk with Conventional forceps	Risk difference with Physics forceps
Adverse events	1752 (10 RCTs)	⨁⨁◯◯ LOW	OR 0.40 (0.25 to 0.64)	137 per 1000	77 fewer per 1000 (99 fewer to 45 fewer)
Adverse events—buccal cortical plate fracture (BCPF)	637 (8 RCTs)	⨁⨁◯◯ LOW	OR 0.42 (0.15 to 1.24)	100 per 1000	56 fewer per 1000 (84 fewer to 21 more)
Adverse events—gingival Laceration	328 (5 RCTs)	⨁◯◯◯ VERY LOW	OR 0.27 (0.08 to 0.93)	201 per 1000	138 fewer per 1000 (181 fewer to 11 fewer)
Adverse events—tooth/root fracture	787 (8 RCTs)	⨁◯◯◯ VERY LOW	OR 0.42 (0.25 to 0.70)	140 per 1000	76 fewer per 1000 (101 fewer to 38 fewer)
Operative time	927 (10 RCTs)	⨁⨁⨁◯ MODERATE	-		MD 20.13 lower (30.11 lower to 10.15 lower)
Pain on first post-operative day	636 (6 RCTs)	⨁◯◯◯ VERY LOW	-	-	SMD 0.81 lower (1.64 lower to 0.03 higher)
* Risk in intervention group (and its 95% confidence interval) based on assumed risk in the comparison group and the relative effect of the intervention (and its 95% CI). CI: confidence interval; OR: odds ratio; MD: mean difference; SMD: standardised mean difference.					
GRADE Working Group grades of evidence High certainty: we are very confident that the true effect lies close to that of the estimate of the effect. Moderate certainty: we are moderately confident in the effect estimate: The true effect is likely to be close to the estimate of the effect, but there is a possibility that it is substantially different. Low certainty: our confidence in the effect estimate is limited, and the true effect may be substantially different from the estimate of the effect. Very low certainty: we have very little confidence in the effect estimate, and the true effect is likely to be substantially different from the estimate of effect.					

## Data Availability

Medline (PubMed) and Scopus databases.

## Appendix A

Search: physics forceps AND extraction

(“physics” [MeSH Terms] OR “physics” [All Fields] OR “physic” [All Fields]) AND (“surgical instruments” [MeSH Terms] OR (“surgical” [All Fields] AND “instruments” [All Fields]) OR “surgical instruments” [All Fields] OR “forcep” [All Fields] OR “forceps” [All Fields]) AND (“extract” [All Fields] OR “extract s” [All Fields] OR “extractabilities” [All Fields] OR “extractability” [All Fields] OR “extractable” [All Fields] OR “extractables” [All Fields] OR “extractant” [All Fields] OR “extractants” [All Fields] OR “extracted” [All Fields] OR “extractibility” [All Fields] OR “extractible” [All Fields] OR “extracting” [All Fields] OR “extraction” [All Fields] OR “extractions” [All Fields] OR “extractive” [All Fields] OR “extractives” [All Fields] OR “extracts” [All Fields])
